# CDCR-Rank: a computational model for predicting drug combination dose response using ranking-based optimization

**DOI:** 10.1093/bioadv/vbag051

**Published:** 2026-05-18

**Authors:** Mohammadamin Moragheb, Karim Abbasi, Parvin Razzaghi, Alireza Dehghan, Sajjad Gharaghani

**Affiliations:** Department of Bioinformatics, Kish International Campus, University of Tehran, Kish, Tehran 3998279416, Iran; Mosaheb Institute for Mathematical Research, Kharazmi University, Tehran 1571914911, Iran; Department of Computer Science and Information Technology, Institute for Advanced Studies in Basic Sciences (IASBS), Zanjan 4513766731, Iran; Department of Computer Engineering, Faculty of Technology and Engineering, Salman Farsi University of Kazerun, Kazerun 7319673544, Iran; Laboratory of Bioinformatics and Drug Design (LBD), Institute of Biochemistry and Biophysics, University of Tehran, Tehran 1417935840, Iran

## Abstract

**Motivation:**

Predicting synergistic drug combinations for cancer therapies is vital but challenged by interaction complexity and vast combinatorial possibilities. To address this, we propose a novel ranking-based approach for drug synergy prediction. Our method builds upon the pre-trained CDCR model to predict absolute synergy scores and introduces a novel architecture to directly compare a list of combinations. A key innovation is our custom loss function, which combines mean squared error with a ranking loss term to simultaneously learn accurate synergy values and their correct relative ordering.

**Results:**

We comprehensively evaluated our model, CDCR-Rank, on the NCI-ALMANAC benchmark under three challenging scenarios, including predicting synergy for novel drug pairs without monotherapy data. CDCR-Rank demonstrated superior performance, consistently outperforming state-of-the-art methods such as comboFM and comboLTR. Ablation studies validated the importance of each component: a one-dimensional convolutional neural network (1D-CNN) for generating task-specific drug representations from SMILES strings, a sinusoidal encoder for modeling non-linear dose-response curves, and the uRank loss for optimizing combination rankings. Our integrated framework achieves significantly improved accuracy and robustness in predicting drug synergy. By reliably prioritizing the most promising combinations for experimental validation, this work has the potential to accelerate the discovery of effective multi-drug cancer therapies.

**Availability and implementation:**

CDCR-Rank is implemented in Python and is available at https://github.com/ParvinRazzaghi/CDCR-Rank

## 1 Introduction

The prediction of drug dose response is a cornerstone of pharmacology and personalized medicine, enabling the identification of optimal dosing that maximizes therapeutic efficacy while minimizing adverse effects (Gundavarapu *et al.* 2024, Bisht *et al.* 2025). This process involves characterizing the fundamental dose-response relationship, typically represented by a sigmoidal curve that reveals critical drug properties like potency and efficacy (Abdel-Rahman and Kauffman 2004, Gumbo *et al.* 2015). However, predicting this relationship is profoundly complex, influenced by a multitude of factors including drug chemical structure, patient genetics, disease state, and interactions with concomitant medications (Tansey *et al.* 2022, Huusari *et al.* 2025). The challenge is further amplified in oncology, where combination therapies are essential to overcome drug resistance and improve patient outcomes. The sheer number of possible drug pairs and dose ratios makes experimental screening, such as that conducted in the NCI-ALMANAC project, both time-consuming and costly (Holbeck *et al.* 2017).

This has motivated the development of computational models to predict drug response and synergy. Early approaches included mathematical formulations to extrapolate combination effects from limited data (Zimmer *et al.* 2016) and methods integrating gene expression with dose-response curves (Goswami *et al.* 2015). Bayesian methods were also introduced to robustly estimate dose-response curves from high-throughput screens (Tansey *et al.* 2022). The field then progressed to machine learning models predicting summary metrics like IC50 or AUC from genomic and chemical properties (Menden *et al.* 2013, Ammad-Ud Din *et al.* 2016). However, a significant limitation of these approaches is their reliance on parametric models as a proxy for true cellular response, making it difficult to discern whether improvements stem from better biological capture or merely better approximation of the proxy model (Rampášek *et al.* 2019).

More recent advances have leveraged deep learning to integrate raw data inputs. Models like DeepSynergy (Rampášek *et al.* 2019) combined drug fingerprints with cell line gene expression profiles using deep neural networks. The problem has also been reframed through different lenses: comboFM applied collaborative filtering, while comboLTR adapted learning-to-rank principles, acknowledging the importance of relative comparison. Parallel research focused on predicting continuous dose-response surfaces (Julkunen *et al.* 2020, Lin *et al.* 2023, Huusari *et al.* 2025). ComboKR, for instance, uses input-output kernel regression to model full response landscapes, demonstrating strong performance for novel drug prediction by requiring only monotherapy data (Huusari *et al.* 2025). Other approaches have employed Bayesian tensor product models (Wheeler 2019) and pharmacokinetic-pharmacodynamic (PKPD) modeling to capture temporal dynamics and tumor growth inhibition (Park 2017).

Despite these advancements, a critical gap remains. Most existing models rely on regression-based loss functions like mean squared error (MSE), which optimize for absolute score accuracy but are agnostic to the relative ordering of predictions (Serafim *et al.* 2023). This is a fundamental misalignment with the primary goal in drug discovery: prioritization. A model can achieve a low MSE while failing to rank the most synergistic combinations highest, severely limiting its practical utility. Furthermore, many models do not fully exploit the structured, dose-dependent nature of synergy data, treating different dose combinations as independent points rather than elements of a rankable list (Xia *et al.* 2018).

To bridge this gap, we propose a paradigm shift toward value-aware ranking optimization. This work introduces CDCR-Rank, a novel framework that integrates a listwise ranking loss directly into the synergy prediction objective. The core insight is that a model must be trained not only to predict accurate synergy scores but also to ensure the correct ordinal relationship between drug-dose combinations.

Ranking-based loss functions are uniquely suited for this task. Unlike traditional losses, they optimize the relative ordering of items, making them ideal for prioritization problems. While pairwise ranking losses consider item pairs and triplet losses use anchor-positive-negative triplets, we employ a listwise ranking loss (uRank) which considers the entire list of combinations simultaneously, thereby capturing the complex, dose-dependent interactions within a full dose-response matrix. This approach enables the model to implicitly learn to identify optimal dose ranges, capture non-linear dose-response relationships, and account for dose-dependent drug interactions—capabilities that are only partially addressed by existing methods.

CDCR-Rank implements this through a multi-modal architecture featuring dedicated encoders: a one-dimensional convolutional neural network (1D-CNN) that learns task-specific molecular representations from SMILES strings, a sinusoidal encoder that captures non-linear, periodic patterns in dose concentrations, and an MLP that projects high-dimensional gene expression data into a latent space. These representations are integrated and trained under a hybrid loss function combining MSE with the uRank listwise loss, ensuring both accurate value prediction and correct combination ranking.

In summary, this work makes the following key contributions:

1. We identify and formalize the critical limitation of regression-oriented loss functions in drug combination prioritization.
2. We propose CDCR-Rank, the first framework to integrate a listwise ranking loss for dose-dependent synergy prediction.
3. We demonstrate through a comprehensive evaluation on NCI-ALMANAC that our ranking-based approach consistently outperforms state-of-the-art methods across multiple challenging scenarios, including predicting synergy for novel drug pairs without monotherapy data.
4. We provide extensive ablation studies validating the importance of each architectural component, with the uRank loss emerging as the primary driver of performance gains.

By aligning the model’s training objective with the practical goal of candidate prioritization, CDCR-Rank provides a more effective and clinically relevant framework for accelerating the discovery of combinatorial cancer therapies.

## 2 Methods

This section first formulates the prediction problem and then details our proposed method.

### 2.1 Problem formulation

In this section, the problem formulation, inputs, and outputs are defined. Assume the training dataset in dose response prediction is shown by ${\left\{(d_{1}^{\left(i\right)},d_{2}^{\left(i\right)},r_{1}^{\left(i\right)},r_{2}^{\left(i\right)},c^{\left(i\right)}),s^{\left(i\right)}\right\}}_{i=1}^{N}$. In this case, the $i^{\mathrm{th}}$ training sample shown by $\left((d_{1}^{\left(i\right)},d_{2}^{\left(i\right)},r_{1}^{\left(i\right)},r_{2}^{\left(i\right)},c^{\left(i\right)}),s^{\left(i\right)}\right)$ where ($d_{1}^{\left(i\right)},r_{1}^{\left(i\right)}$) and ($d_{2}^{\left(i\right)},\;r_{2}^{\left(i\right)}$) respectively show the first and the second drug and its corresponding doses, $c^{\left(i\right)}$ indicates the input cell line. and $s^{\left(i\right)}$ denotes synergy value. The goal is to design a system that takes the input and predicts the synergy value as an output.

### 2.2 Feature encoding

**Figure 1:**
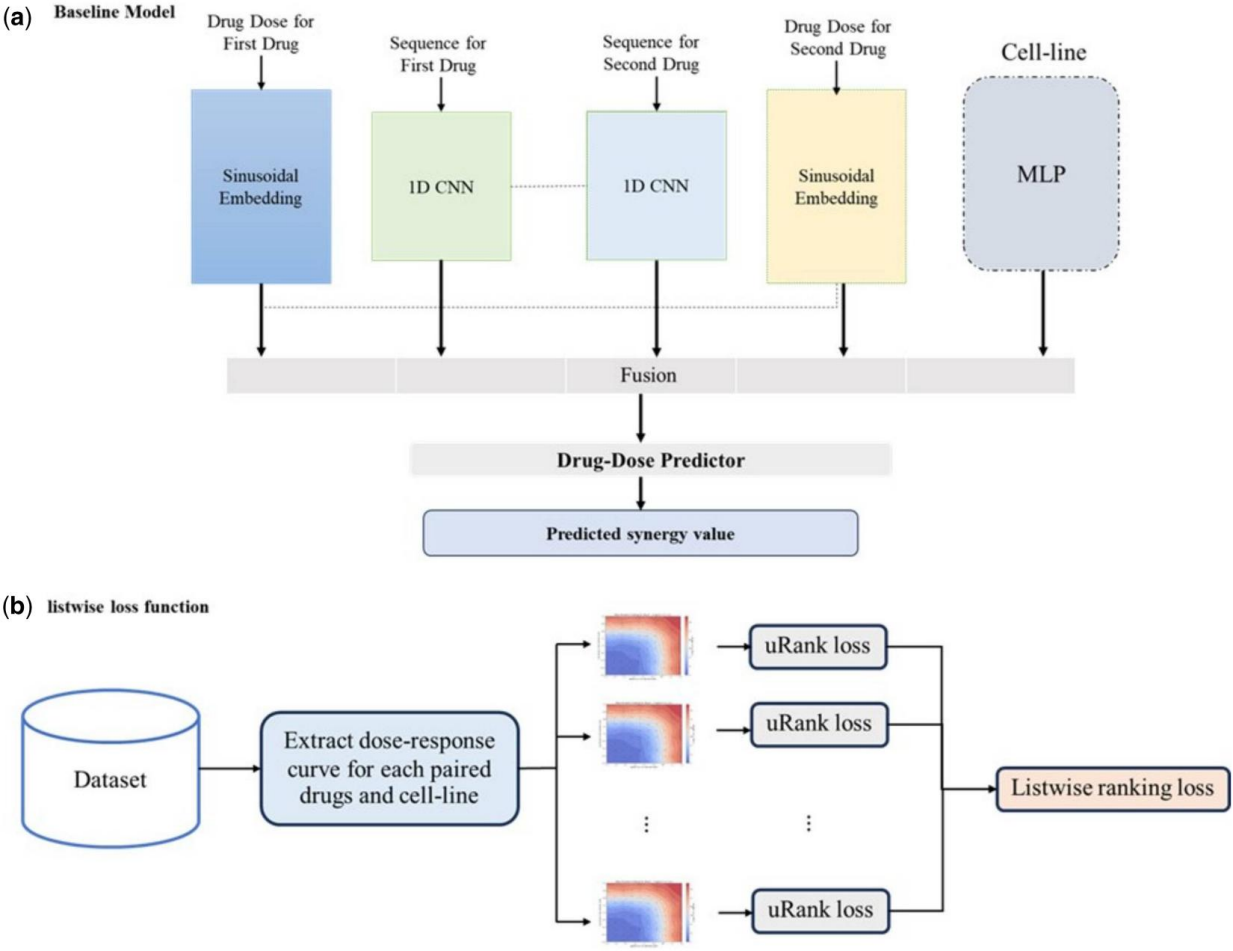
Overall schematic of the proposed methodology. (a) Architecture of the baseline model. (b) Computational workflow for calculating the listwise loss function.

Our research takes a comprehensive approach to predicting the synergy of paired drugs (Fig. 1). We begin by processing the molecular structure of each drug using a 1D-CNN, as shown by $N^{C}$, which maps each SMILES sequence to a 128-dimensional embedding. This architecture processes the sequential nature of SMILES strings to learn features relevant for synergy prediction.

Next, we transform dose values using sinusoidal functions with different frequencies and phases as a dosage feature encoder, producing a 64-dimensional dose embedding, shown by $N^{D}$. This encoding scheme provides a non-linear transformation of dose concentrations that facilitates modeling of dose-response relationships.

In addition to drug and dose features, we process gene expression profiles of cancer cell lines using a multi-layer perceptron (MLP), shown by $N^{G}$. The top 1000 most variable genes across all cell lines are selected and reduced to a 256-dimensional latent representation, preserving information relevant for drug response prediction.

By combining these three components, including drug representations, dose transformations, and processed cell line features, we created a comprehensive input representation that was then fed into a machine learning model to predict the synergy of the paired drugs.

For dose encoding, we employ a sinusoidal transformation to capture non-linear dose-response relationships. Given a dose value r, we compute a 64-dimensional vector as:


(1) $$\text{Encoding}(r,i)=\left\{\begin{array}{cc} \sin\left(r\cdot 10^{-4i/64}\right) & \text{if}\;i\;\text{is}\;\text{even}\\ \cos\left(r\cdot 10^{-4i/64}\right) & \text{if}\;i\;\text{is}\;\text{odd} \end{array}\right.$$

where i=0,1,…,63. This scheme introduces varying frequencies and phases, enabling the model to represent complex, non-monotonic dose-response patterns.

### 2.3 Model architecture

By combining these three components, including drug features, dose representations, and cell line characteristics, we created a comprehensive input representation that was then fed into a machine learning model to predict the synergy of the paired drugs. This integrated approach enabled us to effectively capture the complex interactions between the drugs, doses, and cell lines, ultimately leading to more accurate predictions of drug synergy. Our model was able to learn the non-linear relationships between the input features and the synergy values, and to identify the most important factors that contribute to the synergistic effects of the paired drugs.

The use of a multi-layer perceptron as the prediction model allowed us to leverage the representational power of deep learning architectures, while also providing a flexible and interpretable framework for understanding the relationships between the input features and the predicted synergy values.

Following feature integration, a multi-layer perceptron (MLP) was used as the final prediction model. MLPs have been widely adopted in drug response and drug combination prediction tasks due to their flexibility, robustness, and ability to model non-linear interactions between heterogeneous biological features. Prior studies have demonstrated that MLP-based architectures provide strong and stable performance in similar settings without requiring extensive model-specific assumptions (Ammad-Ud-Din *et al.* 2016, Rampášek *et al.* 2019). In this work, we therefore adopt an MLP as a well-established and reliable baseline learner, allowing us to focus on the impact of the proposed ranking-based optimization strategy rather than model selection.

### 2.4 Implementation details

All experiments were conducted on a server equipped with an NVIDIA RTX 3090 GPU, where the CDCR-Rank model was built and trained end-to-end using the TensorFlow 2.12.0 deep learning framework. The model was trained for 10 000 epochs with a batch size of 256, using the AdamW optimizer configured with a learning rate of 1e-4 and a weight decay of 1e-5.

### 2.5 Loss formulation

In the proposed approach, incorporating the knowledge of drug-dose curves is a crucial aspect of predicting the efficacy of drug combinations. The drug-dose curve represents the relationship between the dose of a drug and its corresponding effect on a biological system. In the context of drug combination therapy, understanding the drug-dose curve is essential to predict the optimal dose of each drug that will result in the desired therapeutic effect. Ranking loss, in this case, can be used to optimize the model’s ability to predict the correct ranking of drug-dose combinations based on their efficacy. By minimizing the ranking loss, the model learns to prioritize the most effective drug-dose combinations and demote the less effective ones. When it comes to training models to predict rankings or scores, one of the key challenges is ensuring that the model is able to accurately capture the nuances of the data. This is where the list-wise ranking loss comes in—a powerful tool that helps to optimize the model’s performance by directly addressing the ranking task.

In essence, the list-wise ranking loss is a type of objective function that measures the difference between the predicted rankings and the actual rankings (Chen *et al.* 2009). But what sets it apart from other loss functions is its ability to consider the entire list of rankings as a whole, rather than just focusing on individual pairs or items.

By taking a holistic approach, the list-wise ranking loss is able to capture the complex relationships between different items in the list and to penalize the model for making mistakes that affect the overall ranking. This is particularly important in applications where the ranking is not just a simple matter of “higher is better,” but rather a nuanced and context-dependent task.

In this approach, we have utilized the uRank model, introduced by Zhu and Klabjan (2020). At first, for each drug pair and cell-line combination, we have extracted all possible tuples with varying doses. Assuming that there are n different measurement points (or dosages) available for these samples, in this case, for these datasets, we have defined a new loss function called uRank loss. Given ${\left\{(d_{1},d_{2},r_{1}^{\left(i\right)},r_{2}^{\left(i\right)},c)\right\}}_{i=1}^{\;n}\;$ denotes these tuples and $S={\left\{s^{\left(i\right)}\right\}}_{i=1}^{n}$ shows the synergy values of these tuples. Assume these tuples are fed into the model to predict the synergy value. The predicted synergy values are shown as follows:


(2) $${{o_{p}^{\left(i\right)}=N^{P}(N}^{D}(d}_{1}),N^{D}(d_{2}),N^{r}(r_{1}^{\left(i\right)}),N^{r}(r_{2}^{\left(i\right)}),N^{c}(c))$$

Where $N^{C}$ as the 1D-CNN drug encoder, $N^{D}$ as the sinusoidal dose encoder, and $N^{G}$ as the MLP-based cell-line encoder.

The algorithm calculates the differences of synergy scores of different tuples with different doses but the same drug pairs and cell-line, which is a key step in the loss calculation process (Algorithm 1: lines 1–2). This is done by creating a matrix P, which contains the differences between the synergy scores of different input tuples:


(3) $$P=Se^{T}-eS^{T}$$

Where a vector e, which is a vector of ones, is used in the calculation to ensure that the loss function is properly normalized. The matrix P is then used to create a mask matrix M, which is used to select the relevant pairs of inputs for the loss calculation:


(4) M={1 if v>0, 0 if v≤0 for v in P}

In mask M, each element indicates if the order between two tuples’ synergy values is correct (0) or incorrect (1). Then the algorithm calculates the exponential of the prediction scores for each input tuple selected by the mask. This is done using the exponential of the prediction scores for each input tuple:


(5) $$L=\ln\left(1+\frac{M.\exp(O)}{\exp\left(O\right)}\right)$$

The matrix multiplication operation, denoted by ·, is used to combine the matrices, while the element-wise division operation, denoted by /, is used to normalize the result. The loss function is then calculated using a combination of matrix multiplication, element-wise division, and Hadamard product operations, involving the matrices and vectors calculated earlier:


(6) $$\mathrm{loss}=\frac{\sum L⊙({\left(2^{s^{\left(i\right)}}-1\right)}_{i=1}^{n})}{n-1}$$

The Hadamard product operation, denoted by ⊙, is used to element-wise multiply the matrices. Overall, the algorithm provides an efficient and optimized way to calculate the loss function, which is essential for training models in tensor-based frameworks. By avoiding loops and using matrix operations, the algorithm can take advantage of the parallel processing capabilities of modern computing hardware, resulting in significant speedups in the training process (see Algorithm 1).

Algorithm 1. uRank loss

**Inputs**: (all possible tuples with the same drug pair and cell-line combination and varying doses)

True synergy value: $S={\left\{s^{\left(i\right)}\right\}}_{i=1}^{n}$

Predicted synergy value: *O*$={\left\{o_{p}^{\left(i\right)}\right\}}_{i=1}^{n}$

**Output:** loss value

**start** 

1   $G={\left(2^{s^{\left(i\right)}}-1\right)}_{i=1}^{n}$

2   $P=Se^{T}-eS^{T}$

3   M={1 if v>0, 0 if v≤0 for v in P}

4   T=M.exp⁡(O)

5   $L=\ln\left(1+\frac{T}{\exp\left(O\right)}\right)$

6   L=L⊙G

7   $\mathrm{loss}=\frac{\sum L}{n-1}$

**return** loss

## 3 Dataset and experimental setup

### 3.1 Dataset

In our research, we utilized the NCI-ALMANAC dataset (Holbeck *et al.* 2017), a comprehensive repository of drug combination dose responses in human cancer cells. This dataset provides a unique opportunity to study the complex interactions between drugs and cancer cells and has been widely used in the field of cancer research.

Gene expression features were selected based on variability across cell lines. We ranked genes by their expression variance and retained the top 1000 most variable genes for model input. This set of genes was used across all 60 cell lines in the study, ensuring a consistent and informative feature space for drug response prediction.

To ensure a robust evaluation of our proposed method, we filtered the data to include only cell lines with a rich set of genetic and molecular characteristics, including gene expression, copy number variation, CRISPR-Cas9 gene deletion, and proteomic profiles. This resulted in a comprehensive dataset containing drug combination and monotherapy response measurements in cancer cell lines from diverse tissue types.

The NCI-ALMANAC dataset is a valuable resource for cancer research, as it provides a systematic and rigorous assessment of how different drug combinations interact with human cancer cells. Each drug combination was carefully screened using a dose-response matrix design, and response measurements were presented as percent growth, adhering to the NCI-60 standard test protocol. Notably, the distribution of combined drug response data across cell lines in our dataset mirrored the distribution observed in the broader NCI ALMANAC study.

In this study, we utilized the NCI-ALMANAC dataset (Holbeck *et al.* 2017), a comprehensive resource of drug combination screening in cancer cell lines. For consistency with prior benchmark studies, we constructed a standardized evaluation dataset following the same filtering criteria used in these comparative methods. Specifically, we selected a subset of 50 FDA-approved drugs from the original NCI-ALMANAC collection, resulting in 617 unique drug pairs. These combinations were screened across all 60 NCI-60 cancer cell lines using dose-response matrices with multiple concentration pairs.

The dataset comprises 333 180 drug combination response measurements and 222 120 monotherapy response measurements, each recorded as percentage growth values following the NCI-60 standard protocol. Gene expression features were processed by selecting the top 1000 most variable genes across all cell lines, ensuring biologically informative representation while maintaining computational efficiency. Drug structures were encoded using standardized SMILES strings retrieved from PubChem (Table 1).

**Table 1:** Summary statistics of the processed NCI-ALMANAC dataset used in this study.

Dataset component	Count	Description/preprocessing
Unique cell lines	60	Filtered for availability of multi-omics profiles (gene expression, copy number variation, CRISPR-Cas9, proteomics) from DepMap.
Unique drugs	104	Standardized SMILES structures retrieved via the PubChem API.
Combination measurements	333 180	Dose-response matrix entries
Monotherapy measurements	222 120	Single-drug responses
Gene features	1000	Top genes selected by the highest variance across all cell lines.

Our approach establishes a robust foundation for further analysis and modeling, enabling the exploration of novel drug candidates and their potential efficacy in specific cancer cell lines. The combination of comprehensive response data, multi-omics characterization, and standardized drug representations sets the stage for more informed and targeted drug discovery efforts.

To evaluate the performance of our model, we employed a five-fold cross-validation strategy, which involved dividing the data into training and testing sets and repeating the process five times. We considered three different scenarios: Scenario S1, where a subset of combination responses from each dose-response matrix was randomly selected and used as the test set; Scenario S2, where a subset of dose-response matrices for specific drug combinations in all cell lines was randomly selected and used as the test set; and Scenario S3, which was similar to S2 but with all monotherapy responses excluded from the training set. This rigorous evaluation framework allowed us to assess the accuracy and robustness of our model and to identify areas for further improvement.

### 3.2 Results

In this section, the obtained results are compared with the state-of-the-art approaches. To ensure a fair and consistent comparison with prior work, all benchmark results reported in this section were obtained using the same dataset configuration, filtering criteria, and evaluation protocols as the corresponding baseline methods. In particular, for comboLTR, comboFM, and comboKR, we adopted the identical subset of the NCI-ALMANAC dataset, including the same set of drugs, cell lines, and dose-response matrices, as well as the same scenario definitions (S1–S3) and cross-validation strategy described in the original studies. Therefore, the performance differences reported in Table 2 reflect methodological improvements rather than variations in data preprocessing or experimental setup. As it is shown, the proposed method could achieve better performance in all three scenarios. The most significant improvement was observed in the third scenario, which is the most challenging. In this case, our method achieved a 3.4% gain over the comboLTR baseline.

**Table 2:** Comparison of the proposed method with recent state-of-the-art approaches under the same dataset configuration and evaluation protocol as reported in the original studies.

Methods	S1	S2	S3
RF	0.923 ± 0.015	0.873 ± 0.009	0.896 ± 0.005
comboFM	0.915 ± 0.012	0.889 ± 0.024	0.878 ± 0.064
comboLTR (28)	0.922 ± 0.011	0.914 ± 0.006	0.915 ± 0.005
comboKR (Huusari *et al.* 2025)	0.900	0.888	
comboKR 2.0 (Huusari *et al.* 2025)	0.902	0.904	0.896
**CDCR-Rank**	**0.954 ± 0.010**	**0.942 ± 0.0008**	**0.949 ± 0.014**

Note: Bold values indicate the best performance achieved for each metric/dataset.

### 3.3 Ablation study

The ablation study is designed to systematically evaluate the contribution of each component in the proposed drug synergy prediction framework. It examines four critical elements: the drug encoder (1D-CNN for SMILES sequences), dose encoder (sinusoidal encoding), and the uRank listwise ranking loss function. For each component, alternative implementations are tested, such as replacing the 1D-CNN with ECFP fingerprints, using linear or logarithmic dose encoding, and substituting the uRank loss with traditional MSE or pairwise ranking losses. This structured approach allows for isolating the individual impact of each architectural choice on overall model performance.

The expected outcomes validate the core hypotheses behind each architectural choice: the 1D-CNN should outperform traditional fingerprints for novel compounds, sinusoidal encoding should better capture non-linear dose-response relationships, cell line-specific encoding should be crucial for accurate predictions across different cellular contexts, and the uRank loss should superiorly optimize drug combination ordering compared to conventional loss functions. The findings not only quantify each component’s contribution but also provide insights for optimizing architecture design in computational drug discovery, potentially leading to more effective machine learning approaches for predicting combination therapies.

The obtained results of the role of the ranking loss in the proposed approach are given in Table 3. As shown, three versions are considered: the first version is the proposed method in which the uRank loss function is removed, and the proposed architecture is learnt only using the standard MSE loss function. In the second version, the uRank loss function is replaced by a pairwise ranking loss function, and the third version is the full proposed method. As shown, the proposed method in all scenarios has better results in comparison to the other versions. It confirms that the uRank loss function could improve model performance. The ablation study on ranking loss functions (Table 3) demonstrates the critical importance of the uRank listwise loss. Compared to traditional MSE loss, uRank improves performance by 5.7%–6.3% across all metrics, while outperforming pairwise ranking loss by 1.8%–4.5%. This substantial improvement validates our hypothesis that listwise optimization better captures the relative efficacy relationships between drug-dose combinations, which is more clinically relevant than predicting absolute synergy values. The exceptionally low variance in scenario S2 (σ = 0.0008) further confirms the robustness of our approach.

**Table 3:** Ablation analysis of optimization objectives.^a^

Method	S1	S2	S3
Study of different ranking loss functions			
Our approach (w.o. uRank loss)	0.897	0.891	0.886
Our approach (w. pairwise ranking loss)	0.936	0.897	0.906
Study of different dose encoding approaches			
Our approach (with linear dose encoding)	0.942	0.939	0.925
Study of different drug encoding methods			
Our approach (with ECFP)	0.908	0.896	0.889
Our approach (with folded ECFP)	0.906	0.898	0.876
Full proposed method			
**CDCR-Rank**	**0.954**	**0.942**	**0.949**

a This table illustrates the performance of CDCR-Rank under different settings: Without uRank, pairwise ranking loss, Linear dose encoding, and with ECFP. Bold values indicate the best performance achieved by the full CDCR-Rank model across all ablation settings.

To evaluate how the dose encoding impacts the final performance of the proposed method, other experiment is done. In this experiment, in the first version, the sinusoidal encoding is replaced with linear dose encoding. In this case, the dose value is fed into a fully connected layer to embed it into a vector. The obtained results are compared with the full proposed method. Our ablation study on dose encoding strategies (Table 3) demonstrates the superiority of sinusoidal encoding over traditional linear encoding. The sinusoidal approach consistently outperformed linear encoding across all evaluation metrics, with improvements ranging from 0.32% to 2.59%. Particularly noteworthy is the exceptional stability achieved with sinusoidal encoding, as evidenced in variance for metric S2 (σ  =  0.0008 versus σ  =  0.015). This enhanced performance and stability can be attributed to the sinusoidal encoding’s ability to capture periodic patterns and non-linear relationships in dose-response data, which are often present in pharmacological interactions but poorly represented by linear transformations.

To investigate how drug encoding impacts the final results, other study was conducted comparing two molecular representation approaches. In the experimental version, the 1D-CNN-based molecular encoder was replaced with Extended Connectivity Fingerprints (ECFP), a traditional cheminformatics representation that captures molecular substructures through circular atom environments. The ECFP fingerprints were generated with a radius of 2 and 1024-bit vectors, then processed through a fully connected layer to match the dimensionality of the original CNN-based embeddings. This setup allowed for direct comparison of traditional fingerprint-based representation against the learned structural features from the 1D-CNN approach. Also, in the second version, instead of using the fully connected layer to map the ECFP because of dimensionality matching, we have utilized a folded ECFP fingerprint. In this case, the folded ECFP fingerprint is directly fed into the MLP for predicting the target variable.

The results, Table 3, demonstrate a substantial performance advantage for the 1D-CNN-based encoding across all evaluation metrics. The full proposed method (CDCR-Rank) outperformed the ECFP-based approach by 5.06% in S1 (0.954 versus 0.908), 5.14% in S2 (0.942 versus 0.896), and 6.74% in S3 (0.949 versus 0.889). This significant performance gap highlights the limitations of fixed fingerprint representations in capturing the complex structural features relevant for drug synergy prediction. Additionally, the 1D-CNN approach showed superior stability, particularly evident in the dramatically reduced variance for S2 (σ  =  0.0008 versus σ  =  0.005).

The superior performance of the 1D-CNN encoder can be attributed to its ability to learn task-specific molecular representations directly from SMILES sequences, capturing nuanced structural patterns and functional group interactions that are particularly relevant for drug combination effects. Unlike static ECFP fingerprints, which employ a fixed molecular representation scheme, the 1D-CNN adapts its feature extraction to the specific requirements of synergy prediction, learning to emphasize molecular characteristics that correlate with combination efficacy. This learnable representation capability proves especially valuable for predicting novel drug combinations where traditional fingerprints may not adequately capture the complex intermolecular interactions that determine synergistic effects.

### 3.4 interpretability framework based on feature attribution

We propose an interpretability framework based on feature attribution and targeted ablation to explain the model’s high-confidence synergy predictions and uncover the biological mechanisms they may represent. This framework moves beyond performance metrics to answer why a specific drug-drug-cell combination is predicted as synergistic.

In attribution analysis via integrated gradients, for a trained model  f, we compute the contribution of each input feature embedding to a given prediction using the Integrated Gradients method (Huusari *et al.* 2025). Given a baseline input $\;x^{\prime }\;$(a zero vector) and the actual input $\;x=(x_{d_{1}},x_{d_{2}},x_{r_{1}},x_{r_{2}},x_{c})$, the attribution for the  i th dimension is:


(7) $${\;\text{Attr}}_{i}(x)=(x_{i}-x_{i}^{\prime })\times \int_{\alpha =0}^{1}\frac{\partial f(x^{\prime }+\alpha (x-x^{\prime }))}{\partial x_{i}}d\alpha \;$$

We compute this for all dimensions of the drug embeddings, dose embeddings, and the cell line embedding.

To quantify the functional importance of attributed features, we conduct a systematic ablation study: first, we identify the top 50 predicted synergistic triplets ($d_{1},d_{2},c$) from the test set, where the maximum predicted synergy score across all dose combinations for that triplet exceeds 0.8. Then, critical feature identification is done. In this case, for each selected high-confidence prediction, we compute attributions and identify critical dimensions for each input modality (Drug 1, Drug 2, Cell Line). A dimension is deemed critical if its absolute attribution value exceeds 60% of the maximum absolute attribution within its modality for that sample. Next, we perform three separate ablation experiments for each high-confidence sample:

- Drug1-ablated: set all critical dimensions of the Drug 1 embedding ($e_{d1}$) to zero.
- Drug2-ablated: set all critical dimensions of the Drug 2 embedding ($e_{d2}$) to zero.
- Cell-ablated: set all critical dimensions of the cell line embedding ($e_{c}$) to zero.

For each ablation, we recompute the synergy prediction. The prediction change (Δ) is defined as the original score minus the ablated score. A large  Δ  indicates that the ablated modality was a primary driver of the synergistic prediction.

This procedure allows us to classify high-confidence synergies as Drug1-dominant, Drug2-dominant, Cell-dominant, or balanced, providing a mechanistic categorization of the model’s predictions.

**Figure 2:**
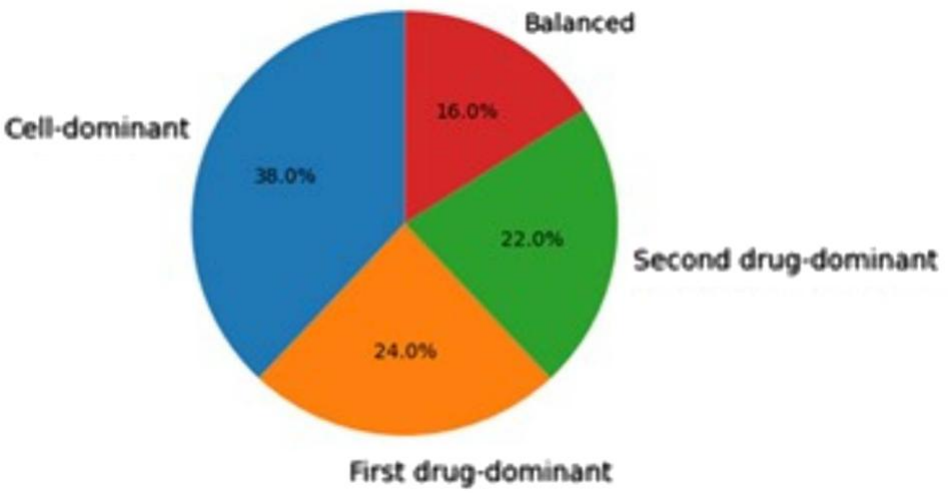
Attribution-guided ablation analysis. Pie chart showing the distribution of the top 50 high-confidence synergy predictions across different driver modalities: Cell-dominant, Drug1-dominant, Drug2-dominant, and Balanced.

To explain the mechanistic drivers behind our model’s predictions, we applied our attribution-guided ablation framework to the top 50 high-confidence synergistic triplets (predicted synergy >0.8). The results, summarized in Fig. 2, reveal distinct patterns in how the model leverages different input modalities.

The ablation analysis classified predictions into three main categories (Fig. 2). In 38% of cases, synergy was Cell-dominant, meaning ablating critical cell line features caused the largest prediction drop ($\Delta_{\mathrm{Cell}}>\Delta_{\mathrm{Drug}1},\Delta_{\mathrm{Drug}2}$). This suggests the model identifies synergies highly dependent on the genomic context, such as specific pathway vulnerabilities present only in certain cell lines. Conversely, Drug1-dominant (24%) and Drug2-dominant (22%) predictions indicate synergies primarily driven by the chemical properties of one agent, potentially representing cases where one drug’s mechanism of action is the key facilitator. The remaining 16% were Balanced, with no single modality’s ablation causing a dominant drop, implying complex, multi-factorial interactions.

## 4 Discussion

The CDCR-Rank framework represents a significant advancement in computational drug combination prediction by successfully integrating multi-modal biological data with a novel ranking-based optimization approach. Our comprehensive experimental results demonstrate that the proposed method outperforms existing state-of-the-art approaches across all evaluation scenarios, with particularly notable improvements in the most challenging setting (S3), where monotherapy responses were excluded from training. This robust performance underscores the model’s ability to generalize to novel drug combinations and cell lines, a critical requirement for practical drug discovery applications.

The key differentiator of CDCR-Rank is not a single component, but the integration of a novel hybrid learning objective within a tailored architecture. We guide the model using a combined loss: MSE loss and uRank loss. The MSE component ensures the predicted synergy scores are accurate in magnitude. The uRank (listwise ranking) component is the crucial addition. It directly optimizes the relative order of all dose combinations for a given drug pair and cell line. It penalizes the model more heavily for misranking a highly synergistic combination than for a small error in its absolute score. This loss forces the model’s encoders (1D-CNN for drugs, sinusoidal for dose, MLP for cell line) to learn representations that are explicitly discriminative for ranking. In other words, this is a different learning signal than merely minimizing the mean error across all data points.

This approach validates a novel paradigm: shifting from pure regression to value-aware ranking optimization. This directly enhances the model’s utility for the real-world task of prioritizing candidate combinations for experimental testing, which we believe constitutes a sufficient advancement for publication.

The ablation studies provide compelling evidence for the importance of each architectural component in the CDCR-Rank framework. The superior performance of the 1D-CNN molecular encoder over traditional ECFP fingerprints (5.06%–6.74% improvement) highlights the value of learning task-specific molecular representations that capture nuanced structural features relevant for drug synergy. Similarly, the sinusoidal dose encoding demonstrated clear advantages over linear encoding, particularly in capturing the non-linear relationships inherent in dose-response data. Most significantly, the uRank listwise loss function proved substantially more effective than both traditional MSE loss (5.7%–6.3% improvement) and pairwise ranking approaches (1.8%–4.5% improvement), validating our hypothesis that optimizing for relative ordering rather than absolute values better aligns with the clinical reality of drug combination prioritization.

Several aspects of our approach contribute to its practical utility in drug discovery. The model’s ability to maintain high performance even when monotherapy data is unavailable (scenario S3) addresses a common limitation in real-world drug screening, where comprehensive monotherapy data may not exist for novel compounds. The exceptional stability demonstrated by the model, particularly the remarkably low variance in scenario S2 (σ = 0.0008), suggests strong reliability for clinical applications. Furthermore, the matrix-based implementation of the uRank loss ensures computational efficiency, making the approach scalable to large-scale drug screening efforts.

However, several limitations and future directions warrant consideration. While the model demonstrates strong performance, the interpretability of deep learning approaches remains challenging. Future work could incorporate attention mechanisms or feature importance analysis to provide biological insights into why specific combinations show synergistic effects. Additionally, the current approach focuses on pairwise drug combinations, while clinical applications often involve higher-order combinations. Extending the framework to handle three or more drugs would significantly enhance its practical utility.

The integration of additional biological data types, such as protein-protein interaction networks or metabolic pathways, could further improve predictive performance by capturing more comprehensive biological context. Similarly, incorporating pharmacokinetic and pharmacodynamic parameters could enhance the clinical relevance of predictions by accounting for temporal dynamics and tissue-specific effects.

In conclusion, CDCR-Rank represents a robust and effective framework for predicting drug combination synergy that outperforms existing approaches while providing insights into the importance of various architectural components. The demonstrated superiority of learned representations over traditional fingerprints, combined with the effectiveness of listwise ranking optimization, provides a strong foundation for future research in computational drug discovery. As the field moves toward increasingly personalized cancer therapies, approaches like CDCR-Rank that can efficiently prioritize promising drug combinations will play a crucial role in accelerating the development of effective treatment strategies.

## Data Availability

CDCR-Rank is implemented in Python, and the dataset is available at https://github.com/ParvinRazzaghi/CDCR-Rank
